# Decitabine Compared with Low-Dose Cytarabine for the Treatment of Older Patients with Newly Diagnosed Acute Myeloid Leukemia: A Pilot Study of Safety, Efficacy, and Cost-Effectiveness

**DOI:** 10.1155/2015/167029

**Published:** 2015-11-04

**Authors:** Linu A. Jacob, S. Aparna, K. C. Lakshmaiah, D. Lokanatha, Govind Babu, Suresh Babu, Sandhya Appachu

**Affiliations:** Department of Medical Oncology, Kidwai Memorial Institute of Oncology, Dr. M. H. Mari Gowda Road, Hombegowda Nagar, Bangalore, Karnataka 560030, India

## Abstract

*Introduction*. The incidence of Acute Myeloid Leukemia (AML) increases progressively with age and its treatment is challenging. This prospective case control study was undertaken to compare the safety, efficacy, and cost-effectiveness of decitabine with those of cytarabine in older patients with newly diagnosed AML who are not fit for intensive chemotherapy.* Materials and Methods*. 30 eligible patients above 60 years old with newly diagnosed AML were assigned to receive decitabine or cytarabine. The primary end point was overall survival (OS). The secondary objective was to compare adverse events and cost-effectiveness of therapy in the two study groups. *Results*. In this study, 15 patients received decitabine and 15 patients received cytarabine. The median OS was 5.5 months for each of the treatment groups. The hazard ratio between the treatment groups was 0.811 with 95% CI of 0.390 to 1.687. Toxicity profile was similar in both groups. Cost per cycle of chemotherapy in INR was 24,200 for decitabine and 1,600 for low-dose cytarabine group. Median of simplified cost-effectiveness ratio was 0.00022 for decitabine group and 0.0034 for low-dose cytarabine group. *Conclusions*. For elderly patients with AML, decitabine and low-dose cytarabine should be chosen based on the patient's choice and affordability. Our study has shown that both of these agents have similar OS and toxicity. Low-dose cytarabine scores over decitabine in developing countries as it is more cost-effective.

## 1. Introduction

The incidence of Acute Myeloid Leukemia (AML) increases progressively with age, from approximately 1 per 100,000 at age 40 to more than 15 per 100,000 at age 75 and older [1]. Unlike younger adults with AML in whom the treatment is straightforward and the goal is cure with intensive chemotherapy, treatment decisions in elderly patients with AML are difficult. Aggressive treatment necessitates hospitalization and separation from family and home, has toxic and potentially fatal side effects, and is often ineffective. The optimal treatment decision for older adults with AML remains highly controversial and is a major challenge for clinicians treating these patients. The clinician has to choose from at least four different approaches: supportive care only, less intensive chemotherapy, standard intensive chemotherapy (IC), or enrolment into a controlled clinical trial. In a developing country like ours, the clinician has to consider the fact that our patients have a higher frequency of poverty, illiteracy, malnutrition, and chronic infectious diseases. Hence, there is a need to explore a treatment strategy which is feasible, affordable, acceptable, accessible, and, more importantly, effective. This study was undertaken to compare the efficacy, safety, and cost-effectiveness of decitabine with those of cytarabine in older patients with newly diagnosed AML.

## 2. Materials and Methods

This was a prospective case control study conducted according to the Declaration of Helsinki after approval by the Institutional Ethics Committee. Between June 2011 and December 2014, patients attending our outpatient department were screened for inclusion in the study. Eligible patients were ≥60 years old with newly diagnosed, histologically confirmed de novo or secondary AML (>20% blasts) who were not fit for intensive chemotherapy with 3 + 7 induction. Exclusion criteria included acute promyelocytic leukemia, inaspirable bone marrow, and HIV infection. Patients must not have had previous chemotherapy (except hydroxyurea) or used experimental drugs for 4 weeks prior to recruitment.

Informed written consent was obtained from all patients prior to protocol entry. A thorough history and physical examination, complete blood count, biochemical profile, chest X-ray, bone marrow aspiration, biopsy, flow cytometry, and cytogenetics were performed at baseline. Patients were assigned to decitabine or low-dose cytarabine depending on the patient's affordability and patient choice guided by the physician. Patients assigned to receive decitabine received 1-hour IV infusion of decitabine 20 mg/m^2^ once a day for five consecutive days every 4 weeks. Patients either were hospitalized or received chemotherapy infusion in daycare department. Patients who opted for cytarabine received 20 mg/m^2^ of cytarabine once a day subcutaneously for 10 consecutive days every 4 weeks. Subcutaneous injections were administered by the primary care physicians in patients' household or patients attenders were trained to give subcutaneous injections. Treatment was continued until death, unacceptable toxicity, lack of clinical benefit, intercurrent illness preventing treatment, or patient request/physician decision to stop. Treatment was delayed at the discretion of the investigator for febrile neutropenia (38.5°C, absolute neutrophil count [ANC] <1,000/*μ*L), clinical and/or microbiologic evidence of infection with grade 3 to 4 neutropenia (ANC <1,000/L), or hemorrhage with grade 4 thrombocytopenia (<25,000 platelets/L). If renal or hepatic dysfunction occurred, treatment was stopped until resolution.

Bone marrow aspiration for response assessment was done only if blood counts normalized with no blasts in the peripheral smear. The primary end point of the study was overall survival (OS). OS was defined as the time from recruitment until death from any cause or last follow-up. The secondary objective was to compare adverse events (AEs) and cost-effectiveness of therapy in the two study groups. All the adverse events during treatment were recorded and graded according to National Cancer Institute Common Toxicity Criteria for Adverse Events (version 3.0) with investigators determining the relationship to the study drug. A simplified cost-effectiveness ratio was calculated in which OS was divided by the cost of the chemotherapy (cost including medicines and daycare charges) multiplied by the number of cycles received.

### 2.1. Statistical Analysis

The patient demographics and baseline clinical characteristics were reported using median and range. Median and range were used to compare number of cycles received and OS. The Kaplan-Meier method was used to describe OS. Analysis of OS was done by log-rank test. Hazard ratios (HRs) and 95% confidence interval (CI) were calculated by using a Cox regression model. The comparison of toxicity between treatments was reported using count (*N*) and percentage (%).

## 3. Results

30 patients were considered for analysis with 15 patients receiving decitabine and 15 patients receiving cytarabine (Table 1). The study observed 12 male patients and 3 female patients in each of the treatment regimes. The mean age was 65, with range of 60–80 years in decitabine group. Similarly, the mean age was 62, with range of 60–73 years in cytarabine group. 86.67% of patients in the decitabine group had de novo AML whereas all the patients in the cytarabine group were de novo in type. Patient demographics and baseline clinical characteristics were well balanced in the two study groups (Table 1). This was a high-risk population as 56% of the patients had ECOG PS 2 and the median blast count in the bone marrow was 40% at baseline.

**Figure 1:**
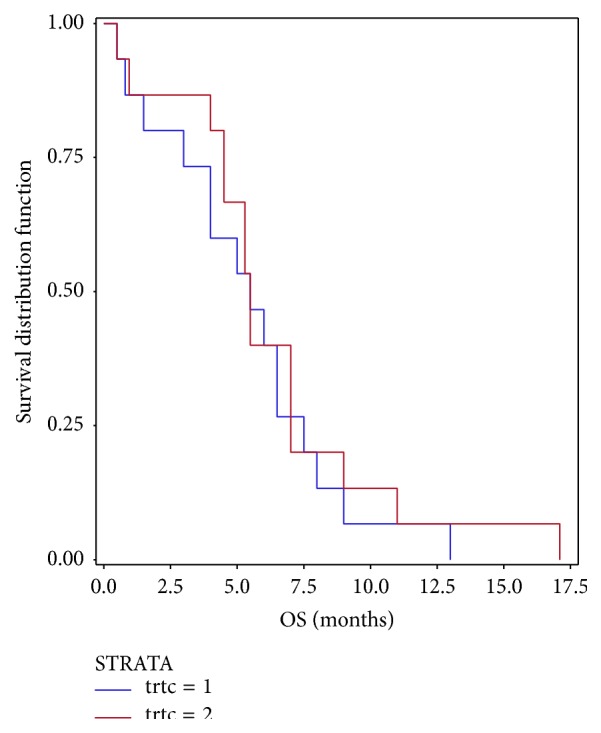
Kaplan-Meier survival curve. Treatment 1: decitabine. Treatment 2: low-dose cytarabine.

The patients in both study groups received a median of 4 treatment cycles (Table 2). The median OS was 5.5 for each of the treatment groups. Based on Kaplan-Meier estimates, the mean OS was 5.38 months and 6.27 months, respectively, for decitabine and cytarabine group. The comparison of survival distribution between treatment groups showed no significant difference at 5% level (Figure 1). The hazard ratio between the treatment groups was 0.811 with 95% CI of 0.390 to 1.687.

It was observed that 2 deaths in each arm were induction mortality. Median OS was 6.0 months and 5.5 months, respectively, for decitabine and cytarabine group after excluding induction mortality patients (Table 3). The comparison of survival distribution after excluding induction mortality patients between treatment groups observed no significant difference at 5% level. The hazard ratio between the treatments was observed as 0.786 with 95% CI of 0.357 to 1.731 excluding induction mortality patients.

Myelosuppression was the most common toxicity observed in both study groups (Table 2). The most common grade 3 and 4 treatment-related AEs with decitabine and cytarabine were thrombocytopenia (decitabine, 53%; cytarabine, 53%), neutropenia (decitabine, 47%; cytarabine, 53%), and anemia (decitabine, 53%; cytarabine, 46%). Febrile neutropenia occurred in 33% of patients in both study groups. Incidence of mucositis, hypokalemia, and hypocalcemia was similar in both study groups.

Cost per cycle of chemotherapy in INR was 24,200 for decitabine and 1,600 for low-dose cytarabine group (Table 5). Median of total cost of therapy was 96,800 and 6,400 for decitabine and low-dose cytarabine group, respectively. Median of simplified cost-effectiveness ratio was 0.00022 for decitabine group and 0.0034 for low-dose cytarabine group.

## 4. Discussion

AML is a common disease of the adult age with a peak incidence between 65 and 70 years. Elderly AML is a clinical entity distinct from the AML in younger adults or children. According to recent epidemiological data, ≥75% of patients with AML are ≥60 years old [2].

In patients above 60 years old, AML is an incurable disease with less than 10% of patients being alive at 2 years [3]. Such a dismal outcome has been traditionally explained by the concurrence of comorbidities and biologically poor-risk AML features. In spite of the above facts, population-based studies have demonstrated that IC prolongs survival and ameliorates quality of life in all age groups, as compared to palliative therapy [4]. Nevertheless, only about one-third of elderly patients receive IC [5].

Cytarabine arabinoside is a pyrimidine analogue which acts by false incorporation into DNA causing reiteration of DNA segments. It also inhibits glycoprotein and glycolipid synthesis, thereby altering membrane structure and antigenicity and thus making tumour cells more prone to natural immune mechanisms. Cytarabine induces synthesis of ceramides and transcriptional factors like junfos and junjun and thus helps apoptosis [6–9]. Cytarabine acts at multiple levels which makes it an essential part of all AML treatment regimens, albeit at different dosages and schedule. It has many advantages like subcutaneous route of administration which eliminates the need for hospitalization and is inexpensive. Low-dose cytarabine has been studied by various authors like Baccarani and Tura [10], Moloney and Rosenthal [11], Housset et al. [12], Kantarjian et al. [13], and Bashir et al. [14] for the treatment of AML (Table 4). Bashir et al. [14] used 20 mg/m^2^ of cytarabine subcutaneously in two divided doses 12 hours apart for 4 days every week for 4 weeks which constituted induction, followed by reassessment. A repeat cycle was administered whenever needed and after attainment of remission, complete or partial. Low-dose cytarabine was continued for 2 days/week as maintenance. 20% of patients achieved complete remission, 30% patients achieved partial remission, and mean duration of survival was 18 months. We chose once-a-day schedule to improve patient compliance and duration of 10 days as used by Kantarjian et al. [13]. In our study, subcutaneous injections of cytarabine were administered by either their primary care physicians or trained attenders of patients. This was convenient for the patients and most patients were compliant with the schedule.

Methylation is involved in silencing the tumor suppressor CCAAT/enhancer binding protein in AML pathogenesis [15]. Decitabine (5-aza-20-deoxycytidine) is a hypomethylating agent with a dual mechanism of action: reactivation of silenced genes and differentiation at low doses and cytotoxicity at high doses. At low “epigenetic” doses, decitabine acts as a hypomethylating agent and inhibits DNA methyltransferase, which appears to have direct cytotoxic effects and/or affect cellular differentiation and apoptosis. In a multicenter, randomized, phase III trial, Kantarjian et al. [13] compared the efficacy and safety of decitabine with treatment choice (TC) in older patients with newly diagnosed AML and poor- or intermediate-risk cytogenetics. Patients (*N* = 485) aged ≥65 years were randomly assigned 1 : 1 to receive decitabine 20 mg/m^2^ per day as a 1-hour intravenous infusion for five consecutive days every 4 weeks or TC (supportive care or cytarabine 20 mg/m^2^ per day as a subcutaneous injection for 10 consecutive days every 4 weeks). The primary analysis with 396 deaths (81.6%) showed a nonsignificant increase in median OS with decitabine (7.7 months; 95% CI, 6.2 to 9.2) versus TC (5.0 months; 95% CI, 4.3 to 6.3; *P* = 0.108; hazard ratio [HR], 0.85; 95% CI, 0.69 to 1.04). Our results are similar to this study with both study groups having a similar OS with no statistically significant difference. Though decitabine is useful in the management of elderly patients with AML, it has many disadvantages like intravenous infusions requiring hospitalization and is very expensive.

“Pharmacoeconomics” is an important topic concerning cancer therapy in the developing countries. Pharmacoeconomics is a scientific discipline that compares the difference in the value of one pharmaceutical drug or drug therapy compared to another for their benefit in a particular health condition [16]. It is a branch of health economics which considers the cost (expressed in monetary terms) and effects (expressed in terms of monetary value, efficacy, or enhanced quality of life) of a pharmaceutical product and estimates the cost : benefit ratio of the drug. Pharmacoeconomic studies are helpful in optimal healthcare resource allocation in resource limited settings. A large number of patients suffering from cancer in India belong to low socioeconomic group. These patients present with advanced stage disease and delay treatment due to the high costs involved. The challenge for resource poor countries like India is to devise treatment strategies which will enable a large number of patients to avail themselves of treatment at affordable costs and obtain a substantial benefit.

From the perspective of a developing country, cytarabine scores over decitabine in terms of its cost-effectiveness, given the equal OS benefit and toxicity profile. Moreover, the induction mortality was also similar in both study groups.

Our study is not without limitations. First, being a nonrandomized study, it is prone to selection bias. Second, the sample size is small and a study with a bigger sample size can only substantiate our observations. Third, response assessment was not done at the end of the first cycle of chemotherapy and remission rates were not documented. Also, as we did bone marrow examination for reassessment only if peripheral blood counts normalized, we could have missed many CRi (complete remission with incomplete blood count recovery). Fourth, quality of life of patients was not considered. Fifth, cytogenetic risk stratification was not done. We are planning a further randomized controlled study keeping the above limitations in mind.

To conclude, our study has demonstrated that, in older patients with newly diagnosed AML who are not fit for aggressive chemotherapy, decitabine and low-dose cytarabine are equally efficacious and have similar toxicity profile. Low-dose cytarabine, being inexpensive and easy to administer as outpatient basis, is more cost-effective and appropriate for resource poor settings. Our study has shown that cytarabine given 20 mg/m^2^/day for 10-day schedule gives acceptable OS and toxicity profile. Low-dose cytarabine could be a safe, acceptable, accessible, and feasible treatment option for elderly AML patients in developing countries.

## 5. Conclusions

Management of AML in older patients is a therapeutic challenge. For patients not eligible for intensive chemotherapy, decitabine and low-dose cytarabine should be chosen based on the patient's choice and affordability. Our study has shown that both of these agents have similar OS rates and toxicity profile. Low-dose cytarabine scores over decitabine in developing countries as it is much less expensive and more cost-effective.

## Figures and Tables

**Table 1:** Patient demographics and baseline clinical characteristics.

Patient characteristics	Decitabine (*N* = 15)	Low-dose cytarabine (*N* = 15)
Age in years (median)	65	62
Sex		
Male, *N* (%)	12 (80)	12 (80)
Female, *N* (%)	3 (20)	3 (20)
Duration of symptoms (in months) (median)	2	1
Type of AML		
De novo, *N* (%)	13 (86.67)	15 (100)
Secondary, *N* (%)	2 (13.33)	0 (0)
Performance score (ECOG), *N* (%)		
1	8 (53.3)	5 (33.3)
2	7 (46.7)	10 (66.7)
Bone marrow blasts, *N* (%)		
20–30%	2 (13.33)	8 (53.3)
30–50%	9 (60)	3 (20)
	4 (26.7)	4 (26.7)
Cytogenetics		
Unsatisfactory	7 (46.7)	8 (53.3)
Normal karyotype	5 (33.3)	6 (40)
Inv(16)	0 (0)	1 (6.7)
Abnormality of chromosome 8	2 (13.3)	0 (0)
Abnormality of chromosome 7	1 (6.7)	0 (0)

**Table 2:** Comparison of outcomes in the study groups.

Outcome	Decitabine (*N* = 15)	Low-dose cytarabine (*N* = 15)
Number of cycles received		
Median	4	4
Range	(1–7)	(1–14)
Overall survival (in months)		
Median	5.5	5.5
Range	(0.5–13)	(0.5–17.10)
Mean survival time	5.38	6.27
Testing equality of survival distribution		
Log-rank test, *P* value	0.5586	
Hazard ratio (95% Wald confidence interval)	0.811 (0.390–1.687)	
Toxicity, *N* (%)		
Febrile neutropenia	5 (33.33)	5 (33.33)
Anemia	8 (53.33)	7 (46.67)
Neutropenia	7 (46.67)	8 (53.33)
Thrombocytopenia	8 (53.33)	8 (53.33)
Mucositis	4 (26.67)	4 (26.67)
Hypocalcemia	3 (20.00)	2 (13.33)
Hypokalemia	2 (13.33)	2 (13.33)
Fatigue	4 (26.67)	5 (33.33)

**Table 3:** Comparison of outcome, excluding induction mortality subjects.

Outcome	Decitabine (*N* = 13)	Low-dose cytarabine (*N* = 13)
Overall survival (in months), excluding induction mortality subjects		
Median	6	5.5
Range	(1.5–13)	(4.0–17.10)
Mean survival time	6.12	7.13
Testing equality of survival distribution		
Log-rank test, *P* value	0.5311	
Hazard ratio (95% Wald confidence interval)	0.786 (0.357–1.731)	

**Table 4:** Different dosage and schedules of low-dose cytarabine.

	Baccarani and Tura [10]	Moloney and Rosenthal [11]	Weh et al. [17]	Kantarjian et al. [13]	Bashir et al. [14]	Present study
Dose of cytarabine	10 mg/m^2^ 12 hourly for 21 days	10 mg/m^2^ 12 hourly for 15 days	10 mg/m^2^ 12 hourly for 14–28 days	20 mg/m^2^ once a day for 10 days	20 mg/m^2^ 12 hourly for 4 days/week	20 mg/m^2^ once a day for 10 days

Overall survival (range)				5 months	18 months (3–24 months)	5.5 months (0.5–17.1 months)

**Table 5:** Cost-effectiveness analysis of chemotherapy in the study groups.

	Decitabine	Low-dose cytarabine
Cost per cycle of chemotherapy (INR)	24,200	1,600
Total cost of therapy (median) (INR)	96,800	6,400
Total cost of therapy (mean) (INR)	95,186	7146
Simplified cost-effectiveness ratio (median)	0.00022	0.0034
Simplified cost-effectiveness ratio (mean)	0.00023	0.0039
